# Evaluation of A Phylogenetic Pipeline to Examine Transmission Networks in A Canadian HIV Cohort

**DOI:** 10.3390/microorganisms8020196

**Published:** 2020-01-31

**Authors:** Lauren Mak, Deshan Perera, Raynell Lang, Pathum Kossinna, Jingni He, M. John Gill, Quan Long, Guido van Marle

**Affiliations:** 1 Department of Biochemistry & Molecular Biology, Alberta Children’s Hospital Research Institute, University of Calgary, Calgary, AB T2N 4N1, Canadathalagalakossinnagep@ucalgary.ca (P.K.);; 2 Department of Medicine, Cumming School of Medicine, University of Calgary and Alberta Health Services, Calgary, AB T2N 4N1, Canada; 3 Department of Medical Genetics, and Mathematics & Statistics, Alberta Children’s Hospital Research Institute, O’Brien Institute for Public Health, University of Calgary, Calgary, AB T2N 4N1, Canada; 4 Department of Mathematics & Statistics, University of Calgary, Calgary, AB T2N 1N4, Canada; 5 Department of Microbiology, Immunology, and Infectious Diseases, Cumming School of Medicine, University of Calgary, Calgary, AB T2N 4N1, Canada

**Keywords:** HIV, Canada, molecular phylogenetics, viral evolution, person-to-person transmission inference, transmission network, summary statistics

## Abstract

Modern computational methods using patient Human Immunodeficiency Virus type 1 (HIV-1) genetic sequences can model population-wide viral transmission dynamics. Accurate transmission inferences can play a critical role in the characterization of high-risk transmission clusters important for enhanced epidemiological control. We evaluated a phylogenetics-based analysis pipeline to infer person-to-person (P2P) infection dates and transmission relationships using 139 patient HIV-1 polymerase Sanger sequences curated by the Southern Alberta HIV Clinic. Parameter combinations tailored to HIV-1 transmissions were tuned with respect to inference accuracy. Inference accuracy was assessed using clinically confirmed P2P transmission patient data. The most accurate parameter settings correctly inferred 48.56% of the P2P relationships (95% confidence interval 63.89–33.33%), slightly lower than next-generation-sequencing methods. The infection date was correctly inferred 43.02% (95% confidence interval 49.89–35.63%). Several novel unsuspected transmission clusters of up to twelve patients were identified. An accuracy trade-off between inferring transmission relationships and infection dates was observed. Using clinically confirmed P2P transmission data as benchmark, our phylogenetic methods identified sufficient P2P transmission relationships using readily available low-resolution Sanger sequences. These approaches may give valuable information about HIV infection dynamics within a population and may be easily deployed to guide public health interventions, without a need for next generation sequencing technology.

## 1. Introduction

The identification and testing of groups at high risk for Human Immunodeficiency Virus (HIV) infection, combined with successfully engaging Persons Living with HIV (PLWH) in combination Anti-Retroviral Therapy (cART) is critical in controlling the HIV epidemic. At the patient level, early clinical intervention and adherence to cART can improve quality-of-life and achieve a near-normal life expectancy [1,2]. At the population level, characterisation of high-risk transmission groups can inform prevention programmes and offer cost-effective intervention strategies [3,4,5,6]. The nature of HIV infection dynamics and the properties of high risk transmission groups in a geographical area, are most granularly described by person-to-person (P2P) transmission relationships [7,8].

Assessing if the infection dynamics in a population is driven by a small number of individuals with repetitive high-risk behaviour or by a larger population with lower frequency risk events allows for the implementation of better-targeted public health interventions and policies. This could be done at a population level without necessarily having to identify specific individuals, thereby minimizing concerns around privacy and marginalization. To provide evidence-based prevention approaches, we need tools that predict P2P transmissions from readily available data. Using HIV sequence data, transmission relationships can be computationally inferred through phylogenetic methods, which compare the genetic similarity of viral sequences to determine the connectivity of HIV transmission in a population [9,10,11].

A key assumption of these methods is that the genetic diversity of within-patient populations is correlated with the time since initial infection [12]. However, the discrepancy between the rates of HIV evolution within and between patients results in inaccuracies from the application of many unified molecular clock assumptions, which are used to estimate phylogenetic tree branch lengths [13,14,15,16,17,18,19]. Furthermore, confounding factors, including; the founder quasispecies, within-patient selective pressure (e.g., cART), immune selection, and technical considerations (e.g., adequate sequencing coverage and data), will affect the estimation of viral evolution observed within a patient [20,21,22,23,24]. As such, the transmission networks obtained from phylogeny-based methods may be inconsistent with what is known about the transmission relationships obtained from well-documented and confirmed clinical data [25,26,27].

At present there are various tools available for HIV transmission analysis, and examples are HIV-TRACE [28] and Cluster Picker [29]. These particular tools are capable of identifying clusters of the viral infection in a cohort but do not provide the direction of infection or the link between direct infection of patients or indirect infection of patients through other (unsampled) individuals situated between source and recipients [28,30]. Moreover, the prediction of HIV transmission clusters using Sanger sequencing requires an individual’s entire viral population to be represented by a single HIV-1 consensus sequence. Due to the lower resolution of Sanger sequences, the genetic differences between the HIV populations of two patients cannot be used to resolve the direction of transmission. Therefore, transmission analysis tools require the date of viral infection, which is typically difficult to pin-point [31]. The objective of the current work was to develop a pipeline that is able to overcome both shortcomings by predicting its own transmission dates as well as predicting unsampled patients to get a better view of the infection dynamics in a particular population.

We describe the development and evaluation of a phylogeny-based analysis pipeline to infer transmission relationships and infection data using routinely collected and easily accessible patient HIV consensus sequences at the Southern Alberta Clinic (SAC). The pipeline consists of rooted phylogenetic tree generation by BEAST2 [32,33] and transmission analysis using TransPhylo [34], compared to thoroughly corroborated clinical infection transmission data. TransPhylo has been previously designed for outbreaks of slowly evolving pathogens such as *Mycobacterium tuberculosis* (MTB) [34]. HIV’s intrinsic rate of mutation is much higher than MTB. However, extensive use of suppressive cART in a population slows the rate of viral evolution, which makes the TransPhylo algorithm suited for analysis of HIV transmission networks with minor modifications [34,35,36,37,38].

Our work shows that using the low resolution consensus sequence data obtained through Sanger sequencing, combined with optimized TransPhylo settings allowed for a good approximation of person-to-person transmissions and novel transmission clusters. We also defined a set of summary statistics based on tree topology that will prioritize candidate transmission trees with the most accurate inferences for analyses where clinical corroboration may be difficult. This pipeline can be easily used and allows one to get valuable insights into the transmission dynamics of a geographically defined epidemic, in clinical settings where next-generation sequencing technologies are not readily available or impractical.

## 2. Materials and Methods

### 2.1. Transmission Pipeline Optimization Overview:

**Figure 1:**
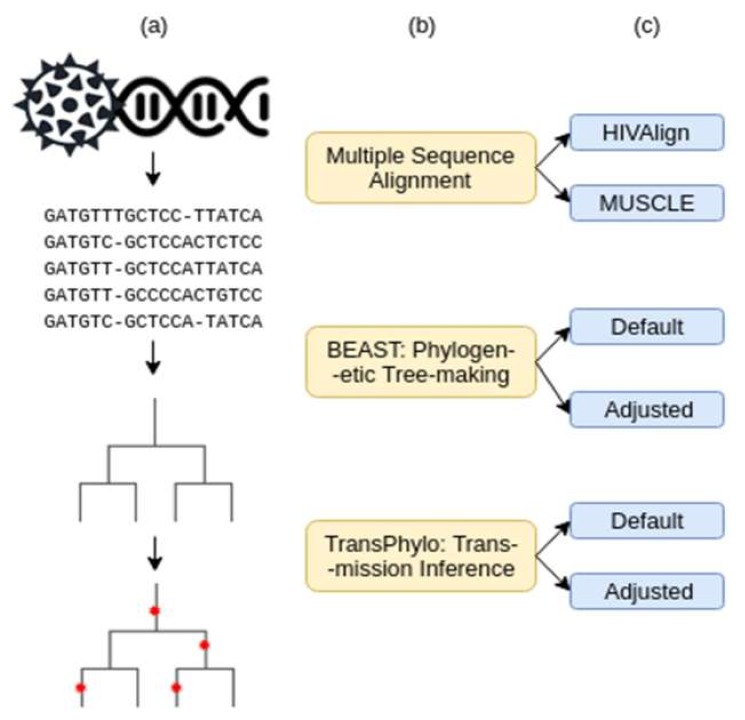
Workflow of data analysis procedures. (**a**) Graphic representation of the data input/output. (**b**) Pipeline tools and how they were used. (**c**) Parameters tested for each tool.

The transmission analysis pipeline consists of three steps: (1) multiple sequence alignment, (2) rooted phylogenetic tree generation, and (3) transmission and infection date inference (Figure 1). Two combinations of parameters at each of the three steps were tested to determine the combination that would provide the most accurate transmission relationship and infection date inferences. These two parameters were dubbed the “Default” and the “Adjusted” parameters. The adjusted parameters were modified to best depict the HIV infection dynamic. Various iterations of adjusted parameters were tested until the best prediction accuracies were reached (Supplementary Tables S2 and S3). The best parameter combination was used as the “Adjusted Parameters”.

In total, eight combinations of parameters were tested. A single transmission tree from each parameter combination was further analyzed for the number of accurate transmission relationships and infection date inferences based on the clinical data. Summary statistics describing the clinical population represented in the transmission tree were calculated for four independent replicates of step 3.

### 2.2. The SAC “Gold Standard” Dataset

The Southern Alberta HIV Clinic (SAC) (Calgary, Alberta, Canada) is relatively geographically isolated and is the single point-of-care clinic for PLWH in Southern Alberta, Canada. This has allowed for comprehensive and longitudinal epidemiologic data to be collected on close to 5000 PLWH since 1989, comprising three decades of data. Currently, 1800 PLWH are active at the clinic. The dataset used in this study was composed of patient HIV sequences and clinical information curated by the SAC database to characterize a sample of the local infection dynamics.

HIV sequences (HIV polymerase (*pol*) region) from 139 PLWH collected as part of routine genotypic antiretroviral drug resistance testing (GART) were used for this study. The patients were selected to model a realistic epidemic, which consist of a mix of clinically reported transmission relationships and infections of unknown origin (detailed in Supplementary Data and Table S1). There were 65 patients selected for which we had thorough clinically reported or confirmed transmission relationships (e.g., known spouse/sexual contact of PLWH or a child of a PLWH). The remainder of the patients were chosen at random from the SAC Database. All clinical patient data has been de-identified. All data is presented as grouped data and analyses cannot be linked back to any individual. The study design, scope of the analyses presented, and the use of this non-nominal data has been approved by the University of Calgary Conjoint Health Research Ethics Board (Ethics ID REB18-2035, original approval 18 December 2018, renewed 18 December 2019).

Patients were chosen for the availability of their GART sequencing data, as well as paired clinical data such as reported transmission relationships and/or HIV testing results. Without paired clinical-sequencing data, it would not have been possible to verify the accuracy of the inferred transmission relationships. Multiple HIV-1 subtypes were examined including subtypes A, B, C, D, G, and several recombinants. Patients with HIV-1 subtype B formed a slim majority of the set, which was expected for a North American epidemic (74/139) [39]. The bulk of the other sequences (51/139) were A, C, and recombinants involving subtype A. Recombinant B/C and subtype D and G, were found in 14 patients (Table S1). GART was performed by the British Columbia Centre for Disease Control/Centre for Excellence in HIV/AIDS GART program (BC CDC/CfE) (Vancouver, British Columbia, Canada). Sequences of HIV polymerase (pol) gene (corresponding to HIV HXB2 genome positions 2526–3544 or 2526–4024) were generated via Sanger sequencing from the GART closest to the estimated infection date and before therapy initiation. The association of HIV sequence data, reported and confirmed transmission relationships and infection dates established a “gold standard” data set to examine the accuracy of the pipeline, and improve the parameter combinations to better reflect the true transmission network in our local situation.

Nucleotide positions associated with drug responses were not removed from the Sanger consensus sequences prior to analysis. However, there is reason to expect that these sequences, though obtained from GART procedures, are largely naive to antiretroviral therapy (ART)-based selection. Prior to 2008, GART was only administered to patients failing ART and pregnant women according to International AIDS Society guidelines. 25/139 (18.0%) of the included patient records had a first-positive HIV test result prior to 2008. As such, the consensus HIV sequence associated with their oldest GART test would have been obtained at treatment failure, significantly later than their date of infection. Although this was not investigated, these reverse transcriptase sequences from these patients may contain resistance mutations since they were obtained at the point of clinical treatment failure [40]. If any of the 25/139 patients took the same ART drugs, their within-host HIV populations would have been exposed to the same selection pressure, and their consensus sequences would have been similar not due to transmission proximity but convergent evolution [41]. In total, however, the HIV sequences associated with 82% of all patients would have been obtained prior to start of ART, so no overt selection pressure due to ART is present for the majority of cases.

Since some of the patients in our cohort were on cART prior to receiving the GART for Sanger sequencing, convergent evolution from similar selective pressures is a concern for 25/139 of the patients [40]. Differences in immune selection based on differences in HLA type amongst patients could potentially be a source of convergent evolution as well [42]. However, the proportion of accurately reconstructed relationships indicates that there are enough patient-lineage-specific mutations that P2P relationships can be identified, without ART or HLA being a confounding factor.

### 2.3. Patient Categorization and Analysis of Inference Accuracy

Patients were divided into three transmission categories (TC; Table 1, Table S1). “Recipients” (*n* = 36) were patients (secondary cases) for whom the source of their HIV had been clinically reported and confirmed. “Donors” (*n* = 29) were patients (primary cases) confirmed to be the source of infection for Recipients. “Controls” (*n* = 74) were patients with no known epidemiological connections, and served as control patients in the data.

**Figure 2:**
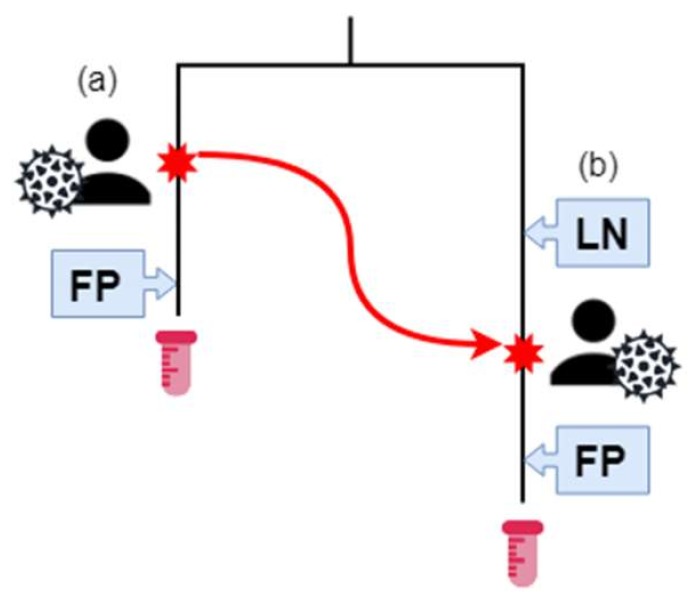
The Southern Alberta HIV Clinic (SAC) patient sequence and transmission data. Patient (A) has HIV, and their estimated infection date is the red star on their branch. They have a positive (First Positive—FP) HIV test result after their infection. At some time-point, represented by a red star and the red arrow, (**a**) infects (**b**). Previously, (**b**) had a negative HIV test. Afterwards, (**a**) has a positive HIV test result. The HIV sequences of samples from (**a**) and (**b**) are phylogenetically related, as represented by the tree. (**b**) is in transmission category 1 and infection category 1. (**a**) is in transmission category 2 and infection category 2. Samples for (**a**) and (**b**) were collected at the end of their respective branches.

Furthermore, we established three infection categories (IC) based on the best estimate of date of infection based on their available Last Negative HIV test (LN) and First Positive HIV test (FP) (Figure 2, Table 1). For those that had a last negative test, the average time frame between last negative and first positive test was 1179 days with a median of 776 days, (range 1–6706 days). IC 1 (*n* = 87) were patients with both LN and FP dates available. IC 2 (*n* = 48) were patients with a documented FP date, but no LN date. IC 3 (*n* = 4) were patients for which no LN or FP dates were available. The majority of patients (28/36) in group Donors were also in IC2. The majority of patients overall (88/139) were associated with both last-negative and first-positive HIV tests. Only three individuals had neither transmission relationship nor infection data associated with their records (Table 1, Figure 2).

### 2.4. Multiple Sequence Alignment

Alignment was accomplished with either a purely distance-based aligner, MUSCLE, or a codon-aware aligner in conjunction with a curated database of homologs, HIVAlign [43,44]. Upon inspection of the output alignments, few indel gaps were observed and the sequences were largely uniform in length. Any differences in length were due to differing coverage during PCR amplification and sequencing approaches.

### 2.5. Parameter Selection

In the selection of the most suitable parameters, the analysis was first conducted using the default BEAST2 and TransPhylo parameters. From the data obtained from these initial parameters, a baseline set of improved, ‘Adjusted’ parameters were determined. The adjusted parameters were designed to better simulate the HIV progression, infection rate and within host evolution in PLWH. The adjusted parameters also took into account the effect of cART and the sampling rates at the SAC (the underlying a priori distribution and assumptions are detailed in Table 2).

### 2.6. Parameter Refinement

Once the baseline-adjusted parameters were determined, their overall accuracy of prediction was tested through 1000 replications of each parameter combination.

TransPhylo considers the generation of the disease and the sampling time as Gamma distributions. Variations to the shape and the scale of the distribution seemed to change the accuracy of prediction for the dates and transmission. By an iterative test all possible combinations for a series of gamma distribution curves were tested. Each combination was replicated 100 times. Then the values with the best results were replicated as before 1000 times and we assessed if there were improved prediction rates compared to the baseline adjusted parameters. In this manner we were able to further refine these two parameters and increase the accuracy for the prediction dates (Tables S2 and S3).

### 2.7. Phylogenetic Tree Generation

Generation of rooted, maximum likelihood (ML) phylogenetic trees from the HIV sequences was accomplished using the program BEAST2 [32,33]. Along with the shape of the tree and the date of the most recent common ancestral sequence of the input sequences, the underlying evolutionary model of the tree was inferred by the Bayesian analysis. Tips were dated with sampling dates provided by SAC. All further transmission inference analysis was conducted on the single ML tree from each run of BEAST2. Two combinations of BEAST2 parameters were used to generate ML phylogenetic trees. The first (“Default”) consisted of the default parameters that would be used for a coalescent population with constant size. The second (“Adjusted”) set of parameters was based on the Bayesian birth–death skyline model, which allows the tree parameters to vary through time in a piecewise fashion (Table 2).

### 2.8. Transmission Relationship and Infection Date Inference

Transmission trees were generated using TransPhylo, a Bayesian program that uses the structure of phylogenetic trees to infer the presence and date of person-to-person transmission relationships as well as the underlying epidemiological parameters [34]. TransPhylo allows for the presence unsampled patients and can assign these unsampled patients in the tree. Sampled patients refer to the 139 patients in the SAC “gold standard dataset”. Unsampled patients refer to any patients not included in the dataset (e.g., they would represent PLWH not in clinical care at the clinic). Both TransPhylo default parameters and adjusted parameters were used to conduct transmission relationship and infection date inferences (detailed in Supplementary Data).

### 2.9. Summary Statistics of the Transmission Tree

The properties of a transmission tree were distilled into a set of seven summary statistics describing the study population. Each value in Table 3 is the average from transmission trees generated by four independent runs of TransPhylo using each of the parameter combinations (detailed in Table 2). These were designed to be compared to known information about the population to prioritize trees likely to contain accurate P2P transmission inferences in cases where gold-standard associations between sequences and P2P transmissions are not available (detailed in Supplementary Data and Table S1).

## 3. Results

The sequences used for this study were obtained from 139 PLWH attending the SAC (Table S1). The HIV transmission risk categories included sexual contact (heterosexual (92, 66%), MSM (men who have sex with men) (33, 24%), and bisexual (14, 10%)), IVDU (intravenous drug use; (11, 8%), being from an endemic area (15, 11%), and maternal foetal transmission (3, 2%). The sex distribution was 88 males and 51 females. The difference between diagnosis dates and dates of sampling were an average of 436 days, with a median of 41 days, and ranging from 4 to 5600 days. The HIV sequences used for analysis consisted of the HIV polymerase (*pol*) region and were collected through Sanger sequencing as part of routine genotypic antiretroviral drug resistance testing (GART). Multiple HIV-1 subtypes were included and consisted of subtypes A (14, 10%), B (72, 52%), C (32, 23%), D (3, 2%), G (8, 6%), and several different recombinants (10, 7%).

PLWH with clinically reported and confirmed transmission relationships as well as a set of individuals with an unknown source of infection were included (described in Table S1.) Transmission and infection dates were inferred based on combinations of the Last Negative (LN) and First Positive (FP) HIV test dates, and were subdivided in three infection groups (IC1, IC2, and IC3; Figure 2, Table 1). IC 1 (*n* = 87) were patients with both LN and FP dates available. IC2 (*n* = 48) were patients with a documented FP date, but no LN date. IC3 (*n* = 4) were patients for which no LN or FP dates were available. In addition, these patients were divided into three transmission categories (TC). These consisted of “Recipients” (*n* = 36), which are patients (or secondary cases) for whom the source of their HIV had been clinically reported and confirmed. “Donors” (*n* = 29) were patients (or primary cases) confirmed to be the source of infection for Recipients. The sequences of the Donor and Recipient groups, were considered the “gold standard data set”. The final group of “Controls” (*n* = 74) were randomly selected patients with no known epidemiological connections, and served as control patients in the data.

### 3.1. Optimizing the Pipeline Parameter Combinations.

The vast majority of sequences supplied to us through the drug resistant testing program were curated and thus lacked indel gaps. Therefore, using either MUSCLE or HIVAlign produced the same multiple sequence alignment, leading to ML (maximum likelihood) phylogenetic trees with similar tree properties and summary statistics (Table 3). Trees from either alignment program with each combination of BEAST2 and TransPhylo parameters were considered technical replicates.

Initially, transmission trees were constructed using default BEAST2 and TransPhylo parameters. The default TransPhylo parameters, in conjunction with the default BEAST2 parameters, predicted nearly three times the number of unsampled patients as source of infection compared to the adjusted parameters. From a clinical perspective, this predicted proportion of unsampled patients (Table 3) was considered unrealistic. Based on Public Health Agency of Canada (PHAC) estimates, 15–20% of PLWH are unaware of their status [45]. Moreover, the default settings predicted the number of sampled patients to be infected after 1989 by another sampled patient (Table 3) at 8–16.25. This estimate was far lower than the number of known P2P transmissions (which was 36 in our data set).

### 3.2. Testing of Multiple Parameters and Development if “Adjusted” Parameters

After extensive testing of multiple parameters, a set of “Adjusted” parameters were obtained (Supplementary Tables S2 and S3). These parameters were selected based on their mean accuracy and 95% confidence interval. The adjusted BEAST2 parameters predicted on average that 27 more patients were infected after 1989 than the default parameters in conjunction with adjusted TransPhylo parameters (Table 3). This was considered more clinically realistic than the default, as all 87 patients with known infection ranges bounded by LN and FP could only have been infected in or after 1992. The default BEAST2 and TransPhylo parameters therefore did not realistically model the transmission dynamics of the local HIV epidemic and did not predict transmission relationships and infection dates accurately (Table 4). We therefore designed the second set of parameters (“Adjusted”) to better reflect the SAC epidemic conditions, as well as HIV genetics. Many of these choices centered on changing the default models for priors to better reflect known properties of the HIV epidemic, such as within-patient evolution. For example, assuming coalescent evolution with a strict molecular clock rate would likely not be an accurate model of HIV within-host evolution, so a relaxed clock model instead of sampling from a log-normal distribution was used. The relaxed model allowed each branch to have its own mutation rate, while the log-normal distribution allowed for most HIV populations (i.e., infections) to diverge from the founder strain at a median rate, and a few to mutate extremely quickly. All BEAST2 and TransPhylo parameters that differ between the Default and Adjusted parameter sets are detailed in Table 2.

### 3.3. Trade-Off between Correctly Inferring Transmission or Infection Dates

We used the Recipients and Donor patients, and the LN and FN data as the benchmark (“gold standard”) for assessing the inference accuracy of each parameter combination. The replicate with the largest number of accurately recovered transmission relationships, 17.48/36 on average, was generated using MUSCLE, adjusted BEAST2 parameters, and adjusted TransPhylo parameters (Table 4). Similarly, accurate replicates were obtained with default BEAST2 parameters (Table 4). However, the replicate with the largest number of accurately inferred infection dates, 37.43 out of 87 on average, was generated using MUSCLE, adjusted BEAST2 parameters, and default TransPhylo parameters (Table 4). Despite the many inaccuracies with regards to transmission inference, the default TransPhylo parameters provided more accurate inferences of infection date. The adjusted parameters demonstrated a tendency to predict infection dates that were older than the patient’s LN. This showed that in choosing TransPhylo parameters, there was a trade-off between transmission relationship and infection date inference accuracy.

### 3.4. Comparing Phylogenetic Inferences with Clinical Epidemiology Data

**Figure 3:**
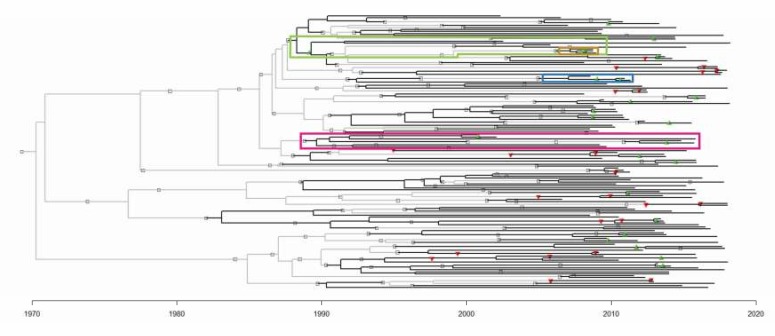
The transmission tree generated by MUSCLE, adjusted BEAST2 parameters, and adjusted TransPhylo parameters. Sampled patients are represented by black branches, and unsampled patients by light grey branches. The symbols represent person-to-person transmission events. Green and red triangles represent correctly and incorrectly inferred relationships respectively. Grey squares represent novel inferences of transmission relationships that have no precedence in the SAC dataset. The branches involved in Figure 4 clusters are highlighted in the correspondingly coloured boxes.

To enhance the accuracy of P2P transmission inference, further analyses of the parameter choices were conducted on a transmission tree replicate generated by MUSCLE, using adjusted BEAST2 parameters, and adjusted TransPhylo parameters. This identified 17.48 of the 36 known transmission relationships (Table 4) [25,27]. For more information about the rooting of the predicted transmission tree (Figure 3) and infection date inference, see the Supplementary Notes and Supplementary Figure S1.

### 3.5. Assessment of The Reliability of Predictions

Using 1000 replications of the eight parameter combinations, we observed that the best results were obtained for disease transmission by the Muscle aligned BEAST2 adjusted and TransPhylo adjusted parameters. For assessing dates of transmission, BEAST2 adjusted and TransPhylo default gave the best results. The BEAST2 adjusted and TransPhylo adjusted had at the highest end 23/36 accurate predictions and at the low end 12/36 accurate predictions at a 95% confidence interval. The highest recorded accuracy for transmission relationships was 26/36 (Seed value: 552) or 72.22%. The accuracy of our Sanger- and BEAST2/TransPhylo-based method is comparable to more complex heuristic-based phylogenetic methods relying on HIV deep sequencing data [25,27]. The infection dates prediction for Adjusted BEAST2 and default TransPhylo had 43/87 correct predictions as the highest and 31/87 correct predictions as the lowest at the same 95% confidence interval. Here too, the most correct predictions were at 47/87 (seed value: 139) or 54.02%. On average, when taking into account the infection dates predicted for each patient at a 95% confidence interval, it was observed that the pipeline predicted the infection date within an average of a 2-year time period.

### 3.6. Assessing Recipients: Secondary Patients in Known Transmission Relationships

Incorrectly inferred transmission relationships were seen in 12/36 patients. Of these, 5/12 were predicted to be infected by another sampled patient. The sequences of these 5/12 patients were placed in closer vicinity with the other sampled patients than the actual source, leading to the incorrectly inferred relationship. Thus, using BEAST2 ML estimates of genetic similarity failed to recapitulate transmission 5/36 times. Seven out of twelve were predicted to be infected by an unsampled patient instead of the actual source. For these 7/12 patients, the within-host process modelled by TransPhylo incorrectly assumed the presence of an unsampled patient. Thus, using a simple model of coalescent evolution failed to recapitulate transmission 7 out of 36 times.

### 3.7. Assessing Donors: Primary Patients in Known Transmission Relationships

For Donors, 10/29 were incorrectly predicted to be infected by their corresponding Recipients secondary patients. For example, if it was known that A infected B in the SAC dataset, then TransPhylo instead predicted a B-to-A infection direction. All 10 incorrect inferences occurred because of incorrectly inferred infection dates, either for the Donors or the Recipients patient. Again, this demonstrates that the trade-off between correct transmission inferences comes at the expense of accuracy of the infection date inference. However, many of the correctly inferred transmission relationships highlighted in the Recipients analysis occurred despite the predicted infection dates falling outside of a viable infection date range. It is thus possible to identify person-to-person transmission without identifying exactly when it happened, and these novel transmission inferences may in fact have occurred.

### 3.8. Identification of Novel Transmission Clusters

**Figure 4:**
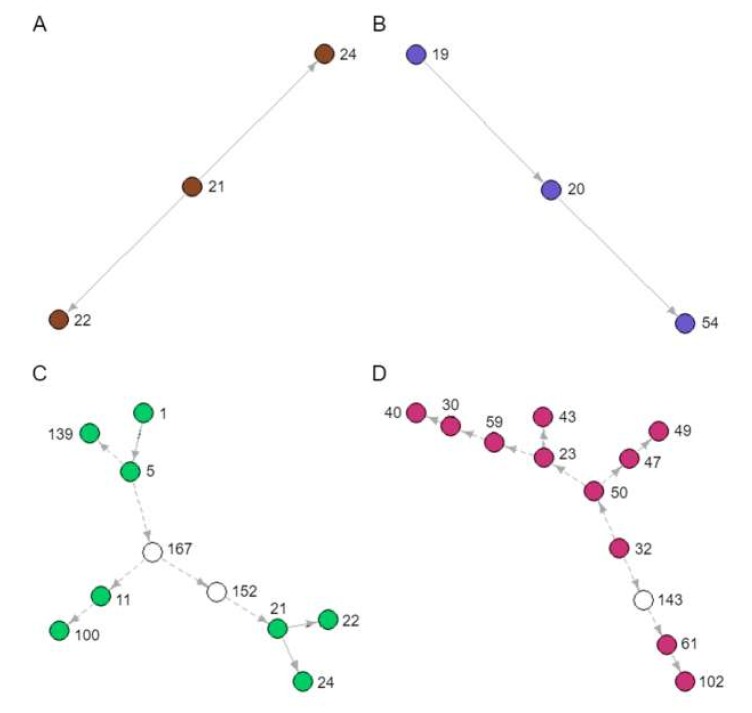
Novel transmission clusters (**A**, **B**, **C** and **D**) identified from the transmission tree in Figure 3. Each node represents a sampled patient from the SAC database, and each arrow a directed transmission relationship. Coloured nodes represent sampled patients and indicate the corresponding coloured boxes in Figure 3. White nodes represent predicted unsampled patients. Solid arrows represent transmission relationships already known to the SAC, whereas dashed arrows represent novel inferences of transmission.

During our analyses, several novel and unknown transmission clusters were predicted (Figure 4). The tree branches involved in each cluster are indicated in Figure 3. They ranged in size from 3 to 12 people. Figure 4A,B depicts clusters of sampled patients already in known transmission relationships (Recipients and Donors), whereas Figure 4C,D is largely composed of novel inferences (Recipients-3). The 12-patient cluster was predicted to span 16 years of infection history (Figure 4D). When we looked back at the data captured in the clinic database for these patients, there are actually public health contact and blood transfusion trace back indicators that would support that these are likely credible transmission relationships. However, further in depth trace back analysis will be required that falls beyond our ethics approval. There were numerous instances of sampled patients infecting unsampled patients, especially earlier than 1989, when the SAC started operation (Figure 4C,D). These unsampled patients were predicted by the model to have been the source of HIV for sampled patients in later years.

## 4. Discussion

We demonstrated the ability of a phylogeny-based analysis pipeline to correctly infer transmission relationships and infection dates from routinely collected low resolution Sanger derived HIV consensus sequences of HIV infected individuals, as well as an ability to identify novel unsuspected transmission clusters. The transmission-optimized parameter combination (adjusted BEAST2 and adjusted TransPhylo) was able to recover on average, 48.56% of clinically reported person-to-person transmission relationships with a high of 63.89% and a low of 33.33% at a 95% confidence interval. Despite the presence of pol sequences from other randomly selected SAC patients and without pinpointing infection dates accurately, the pipeline still identified the direction of transmission in a majority of verifiable relationships. Of note, 22.2% of the verifiable P2P instances still involved the correct pair of patients, but just had the wrong direction of transmission. The correct patient pair was identified by genetic similarity, but the within-patient evolution model predicted an erroneously earlier infection date for the secondary (recipient) patient. Our pipeline uses solely Sanger sequenced data of one HIV region (*pol*) and less computational intense approaches. Despite this, it performs quite close to the more expensive NGS based prediction pipelines combined with extensive mathematical models and more complete HIV genome sequences [31]. It should also be noted that we used what we called a “gold standard dataset”, which was confirmed by thorough and extensive public health tracing and followed up by the Southern Alberta Clinic, whereas some other work on assessments of prediction accuracy did not have access to similar thorough “gold standard” datasets [31]. As a follow up, integrating within-host HIV diversity in the models [46] as well as extending to sequences beyond the HIV polymerase region will allow for further fine tuning of the pipeline, and increase its predictive power. With these additions, the pipeline will be able to be used in low resource settings. We also encourage researchers to perform these analyses in their settings with readily available data sets to examine how well the pipeline can be applied to different settings and recapitulate the HIV infection dynamics.

With different combinations of parameters, there was a small but replicable improvement in the accuracy of infection date inference, but at the expense of predicting the correct transmission relationship. There are currently no algorithms flexible enough to infer an integrative evolutionary and epidemiological model explaining within and between patient HIV genetic variability. HIV subtype did not affect inference accuracy, but other biological and technical considerations may still have a role. The parameters underlying within and between patient evolution have been shown to be different [12,13,14,15,16,17]. This variability could have been exacerbated by technical procedures that amplify only the most frequent variant. Since cART is widely and intensely used in the SAC patient population, convergent evolution from selective pressures of cART at the population level is possible. However, the proportion of accurately reconstructed relationships indicates that there are enough patient-lineage-specific mutations that P2P relationships can be identified. One way to circumvent complications of genetic similarity would be to compare transmission inferences from other genes, such as envelope or other group-specific antigens.

In the absence of even more precise “gold standard” datasets such as the one used in this study, P2P transmission is generally difficult to verify. Thus, we have developed summary statistics that can be used to prioritize transmission trees for further analysis based on their agreement with verifiable transmission event information based on clinical and patient reporting within the affected populations. Though these are not direct indicators of accurately inferred transmission relationships and infection dates, they are useful in identifying the parameter combinations that best describe the underlying transmission dynamics of the population of interest.

The closer the summary statistics are to clinical predictions of the PLWH population, the better the parameter combination was at recovering accurate transmission relationships, at the expense of predicting a larger proportion of false positives (Table 3 and Table 4). With adjusted TransPhylo parameters, the average number of sampled patients predicted to be infected after 1989 by another sampled patient (Table 3) was much larger than the number of known sampled-to-sampled transmissions (*n* = 36). The difference between the estimated and actual numbers consisted of infection relationships between the right patient pair but in the wrong direction (for example, predicting (b) → (a) transmission in Figure 2, with the actual relationship being (a) → (b), but also included novel transmission predictions between patients (for example, (a) → unsampled patient). The more unsampled patients predicted in the tree, the better the parameter combination was at predicting infection dates that fell in viable infection ranges, but simultaneously, worse at predicting P2P transmissions. The estimated ratio of multiple-to-single secondary patients was also calculated to determine how well TransPhylo captured the relative risk of being infected by PLWH engaging in high-risk behaviour, resulting in multiple secondary infections [40,41]. The ratio was significantly higher with adjusted BEAST2 parameters than the default. There was a relative increase in the number of multiple-secondary-case patients when the adjusted TransPhylo parameters are applied. Since transmission trees predict not only P2P relationships but the directionality of entire novel transmission clusters, the epidemiological properties of especially high-risk groups can be described. Parallel efforts involving simulated epidemics indicate that implementation of prevention strategies targeted at high-risk groups reduce the rate of cluster growth [47,48]. From a public health standpoint, an ability to characterize the epidemic at a person-to-person level would help determine if the epidemic is being driven by a small number of individuals with repetitive high-risk behaviour or by a larger population with lower frequency risk events. Such information would help target public health interventions and policies. However, this does raise both an ethical and potential legal concern, with regard to identifying directionality of transmission. Utmost care needs be taken and the data needs to be anonymized so only risk groups can be identified and not specific individuals. This approach can give insight and better estimates of numbers of infected individuals not in care out in the community, which helps to inform overarching public health interventions (i.e., testing, prevention and educational programs), while avoiding marginalizing any of the risk groups identified through these types of analyses.

## 5. Conclusions

We demonstrate that consensus polymerase sequences from routine clinical Sanger sequencing alone achieves robustness at inferring transmission relationships and infection dates [25,27,49]. Our approach can be straightforwardly implemented in settings where NGS is not readily available. In addition, it allows for the analysis and use of historical clinical and Sanger data already available in many clinical settings. Applying these analyses to a variety of settings can provide valuable information about population-wide HIV infection dynamics and a detailed understanding of local HIV epidemics, which provides an opportunity for more guided public health interventions.

## Figures and Tables

**Table 1:** Breakdown of the number of patients based on their clinical data completeness.

		Transmission Categories (TC)			Total
		Recipient	Donor	Control	
**Infection Category (IC)**	**1**	28	14	45	87
	**2**	8	14	26	48
	**3**	0	1	3	4
	**Total**	36	29	74	**139**

**Table 2:** Comparison of default and adjusted BEAST2 TransPhylo parameter combinations, and rationale for the choice of adjusted parameter values.

Analysis Tool	Parameter Description	Default Model(s)	Adjusted Model(s)	Rationale
**BEAST 2**	Molecular clock model	Strict clock model	Uncorrelated relaxed clock, rates drawn from log-normal distribution	By relaxing the clock and allowing non-zero clock variance, each branch can have its own rate of mutation. Better modelling of variable within-patient selective pressure, especially over multiple eras of combined antiretroviral therapy ranging from the 80s to the 10s can be obtained.
	Substitution model	Hasegawa, Kishino and Yano (HKY) model	General time-reversible (GTR) model	By allowing the rate of each base-to-base substitution to be estimated independently, as opposed to just transitions and transversions, the overall mutational process can be estimated more accurately (especially for a highly mutable virus like HIV).
	Population growth model	Uniform size for coalescent-only populations	Birth-death skyline serial model	The generation, sampling, and removal time distributions are estimable. By allowing for arbitrary changes in the effective population size of HIV across patients, the dynamics of non-coalescent transmission histories can be modelled more accurately.
**TransPhylo**	Likelihood that a source is sampled	0.5, unfixed	0.99, fixed	If the likelihood is left unfixed, TransPhylo predicts nearly 3.6× as many patients. As the SAC population is i) relatively small, ii) geographically isolated and iii) the sole HIV care provider, having so many HIV-positive patients unknown to the SAC clinic is unlikely.
	Generation time distribution	Shape = 2 Scale = 1.5 Both unfixed	Shape = 2 Scale = 1 Both unfixed	A slight reduction in the initiating scale parameter, increasing the amount of time between new infections, seems to better infer transmission relationships.
	Sampling time distribution	Shape = 2 Scale = 1.5 Both unfixed	Shape = 2 Scale = 1 Both unfixed	A slight reduction in the initiating scale parameter, increasing the amount of time between infection and sampling, seems to better infer transmission relationships.

**Table 3:** Summary statistics describing transmission trees generated by each combination of parameters for each program.

Aligner	MUSCLE				HIVAligner			
BEAST	Default		Adjusted		Default		Adjusted	
TransPhylo	Default	Adjusted	Default	Adjusted	Default	Adjusted	Default	Adjusted
**Patients in Tree ^A^**	813.25	228.25	640.75	178.25	810.5	222.75	620.75	173.25
**Sampled Infected >1989 ^B^**	139.5	109.25	140	137	139.75	110.5	140	137.5
**Infected by Sampled Patient ^C^**	8.75	55	12	85	8	54.5	16.25	84.75
**Unsampled,** **Infected >1989 ^D^**	318.25	17.75	459.75	27.25	342.25	17.5	446.5	25.75
**Proportion ^E^**	0.063	0.503	0.086	0.620	0.057	0.493	0.116	0.616
**Proportion ^F^**	0.695	0.140	0.767	0.166	0.710	0.137	0.761	0.158
**Ratio of Multi-to Single-Infectors ^G^**	0.113	0.154	0.228	0.332	0.119	0.162	0.227	0.411

(^A^) The total number of patients represented in the tree. (^B^) The number of sampled patients predicted to be infected after 1989. (^C^) The number of (^B^) that was predicted to be infected by another (^B^). (^D^) The number of unsampled patients predicted to be infected after 1989. (^E^) Proportion of sampled patients that were infected by other sampled patients. (^F^) Proportion of patients predicted to be infected after 1989 that are unknown to the SAC. (^G^) Ratio of patients who were predicted to have infected multiple people to those who were predicted to have infected a single person.

**Table 4:** The average of accurately inferred person-to-person (P2P) transmission relationships and infection dates in selected transmission trees generated by each combination of parameters for each program over 1000 replications at their 95% confidence.

Align	BEAST	TransPhylo		Transmission Relationships				Infection Dates				
			Avg	Low 2.5	Up 97.5	Diff	Total	Avg	Low 2.5	Up 97.5	Diff	Total
**MUSCLE**	**Adjusted**	**Adjusted**	**17.477 ** *	12	23	11	36	22.456	17	27	10	87
		**Default**	6.445	3	11	8		**37.434 ** *	31	43	12	
	**Default**	**Adjusted**	15.855	10	21	11		18.45	14	23	9	
		**Default**	3.679	1	8	7		30.696	25	36	11	
**HIV Aligner**	**Adjusted**	**Adjusted**	17.461	12	23	11		22.346	18	27	9	
		**Default**	6.16	2	10	8		37.089	31	44	13	
	**Default**	**Adjusted**	15.914	11	22	11		18.411	14	23	9	
		**Default**	3.714	1	7	6		30.867	25	36	11	

* The largest number of correctly inferred transmission relationships and viable infection dates are in bold and underlined.

## Supplementary Materials

The following are available online at https://www.mdpi.com/2076-2607/8/2/196/s1, All clinical patient information analyzed in this manuscript was de-identified prior to analysis and can be found in Table S1. The HIV sequences from each of the patient samples are available upon request from the investigators. The following supplementary data are available online. Figure S1: Transmission tree generated by HIVAlign, default BEAST parameters, and adjusted TransPhylo parameters, Table S1: Inferred transmission relationships and infection dates, Table S2: Actual parameter values and prior settings of default and adjusted parameter sets for BEAST, Table S3: Summary statistics of parameter testing for TransPhylo Shape and Scale parameter testing for infection and sampling rate.

## All Codes for the Analyses can be Found Under

Project name: Transmission Analyzer, Project home page: https://github.com/heLongLab/Transmission_Analyzer, Operating system(s): Platform independent, Programming language: R version 3+, License: MIT License, no restrictions for usage.

## Abbreviations

HIV	Human Immunodeficiency Virus
P2P	Person to Person
cART	Combination Antiretroviral Therapy
SAC	Southern Alberta HIV Clinic
MTB	*Mycobacterium tuberculosis*
PLWH	Persons Living With HIV
*pol*	Polymerase
GART	Genotypic Antiretroviral Drug Resistance Testing
LN	Last Negative HIV Test
FP	First Positive HIV Test
TC	Transmission Category
IC	Infection Category
PHAC	Public Health Agency of Canada
