# Supplementary material for: Evaluation of A Phylogenetic Pipeline to Examine Transmission Networks in A Canadian HIV Cohort
Source: Microorganisms. 2020 Jan 31;8(2):196. doi: 10.3390/microorganisms8020196 (PMC7074708; doi:10.3390/microorganisms8020196)
Supplement: Supplementary file 1 [file microorganisms-08-00196-s001.zip › Mak_ etal_Table_S1.docx]

| **Patient ID^A^** | **Clade^B^** | **Trans. Ev.^C^** | **Trans. Acc.^D^** | **Infect. Ev.^E^** | **Infect. Acc.^F^** | **Presumed Source^G^** | **Predicted Source^H^** | **Last Negative Date^I^** | **First Positive Date^J^** | **Pred. Infect. Date^K^** |
| --- | --- | --- | --- | --- | --- | --- | --- | --- | --- | --- |
| 1 | B | 1 | 3 | 2 | 0 | NA | 170 | Jan-92 | Jan-93 | Oct-88 |
| 2 | C | 0 | 3 | 0 | 3 | NA | 169 | None | None | May-94 |
| 3 | B | 1 | 3 | 1 | 1 | NA | 104 | None | May-96 | Apr-95 |
| 4 | B | 2 | 1 | 2 | 0 | 3 | 3 | Jan-97 | Dec-01 | Jan-02 |
| 5 | B | 2 | 1 | 2 | 0 | 1 | 1 | Feb-00 | Feb-02 | Apr-90 |
| 6 | CRF01_AE | 0 | 3 | 1 | 0 | NA | 112 | None | Jan-02 | Apr-03 |
| 7 | CRF02_AG | 0 | 3 | 2 | 0 | NA | 58 | Oct-00 | Dec-02 | Dec-04 |
| 8 | B | 0 | 3 | 1 | 1 | NA | 164 | None | Oct-03 | Jun-99 |
| 9 | B | 0 | 3 | 2 | 0 | NA | 1 | Jan-02 | Oct-04 | Dec-96 |
| 10 | C | 1 | 3 | 1 | 0 | NA | 162 | None | Jan-03 | May-03 |
| 11 | B | 0 | 3 | 2 | 0 | NA | 167 | Jun-05 | Sep-05 | Apr-00 |
| 12 | A1 | 1 | 3 | 1 | 0 | NA | 156 | None | Jan-98 | Oct-02 |
| 13 | A | 0 | 3 | 2 | 0 | NA | 165 | Jan-04 | Apr-06 | Jul-02 |
| 14 | B | 0 | 3 | 1 | 1 | NA | 159 | None | Jun-07 | Apr-95 |
| 15 | B | 0 | 3 | 1 | 1 | NA | 101 | None | Jan-08 | Dec-99 |
| 16 | C | 1 | 3 | 1 | 1 | NA | 168 | None | Jan-04 | Mar-96 |
| 17 | B | 1 | 3 | 2 | 0 | NA | 145 | Jan-06 | Jan-07 | Jan-12 |
| 18 | G | 0 | 3 | 2 | 0 | NA | 175 | Jan-04 | Jan-06 | Mar-83 |
| 19 | B | 1 | 3 | 2 | 0 | NA | 157 | Oct-06 | Jul-08 | Feb-06 |
| 20 | B | 2 | 1 | 2 | 0 | 19 | 19 | Jan-05 | Jul-08 | Mar-10 |
| 21 | B | 1 | 3 | 1 | 1 | NA | 152 | None | Jul-08 | Apr-07 |
| 22 | B | 2 | 1 | 2 | 0 | 24 | 21 | Jan-06 | Sep-08 | May-09 |
| 23 | B | 0 | 3 | 0 | 3 | NA | 50 | None | None | Feb-01 |
| 24 | B | 2 | 1 | 1 | 0 | 21 | 21 | None | Nov-08 | Mar-09 |
| 25 | CRF03_AB | 0 | 3 | 1 | 1 | NA | 93 | None | Feb-09 | Nov-94 |
| 26 | C | 1 | 2 | 1 | 0 | NA | 41 | None | Jan-04 | Nov-06 |
| 27 | B | 0 | 3 | 2 | 0 | NA | 172 | Jan-91 | May-09 | Mar-89 |
| 28 | C | 2 | 1 | 2 | 0 | 26 | 41 | Jun-00 | Jun-04 | Aug-14 |
| **Patient ID^A^** | **Clade^B^** | **Trans. Ev.^C^** | **Trans. Acc.^D^** | **Infect. Ev.^E^** | **Infect. Acc.^F^** | **Presumed Source^G^** | **Predicted Source^H^** | **Last Negative Date^I^** | **First Positive Date^J^** | **Pred. Infect. Date^K^** |
| 29 | B | 1 | 3 | 2 | 0 | NA | 159 | Jan-06 | Jan-08 | Nov-05 |
| 30 | B | 1 | 3 | 1 | 0 | NA | 59 | None | Jan-01 | Oct-07 |
| 31 | B | 0 | 3 | 1 | 0 | NA | 45 | None | Jan-01 | Oct-09 |
| 32 | B | 0 | 3 | 2 | 0 | NA | 159 | Jan-07 | Jan-10 | Sep-91 |
| 33 | B | 1 | 3 | 2 | 0 | NA | 163 | Jan-05 | Jan-09 | Mar-03 |
| 34 | B | 0 | 3 | 1 | 1 | NA | 39 | None | Jan-04 | Sep-97 |
| 35 | B | 0 | 3 | 2 | 0 | NA | 164 | Jan-03 | Apr-09 | Jun-93 |
| 36 | D | 0 | 3 | 2 | 1 | NA | 149 | Jan-08 | Dec-09 | Jul-09 |
| 37 | B | 1 | 2 | 1 | 1 | NA | 81 | None | Apr-10 | Feb-10 |
| 38 | G | 0 | 3 | 2 | 0 | NA | 18 | Jan-08 | Apr-10 | May-89 |
| 39 | B | 0 | 3 | 2 | 0 | NA | 35 | Jan-99 | Nov-05 | Nov-95 |
| 40 | B | 2 | 1 | 2 | 1 | 30 | 30 | Aug-09 | Mar-10 | Dec-09 |
| 41 | C | 2 | 0 | 1 | 1 | 28 | 138 | None | Feb-10 | Oct-98 |
| 42 | CRF01_AE | 0 | 3 | 1 | 1 | NA | 112 | None | Jun-10 | Dec-03 |
| 43 | B | 0 | 3 | 1 | 1 | NA | 23 | None | Oct-08 | Dec-06 |
| 44 | B | 0 | 3 | 1 | 1 | NA | 69 | None | Aug-09 | Mar-05 |
| 45 | B | 1 | 3 | 2 | 0 | NA | 154 | Jan-08 | Sep-10 | Mar-06 |
| 46 | B | 2 | 1 | 2 | 0 | 45 | 45 | Apr-10 | Oct-10 | Dec-10 |
| 47 | B | 1 | 3 | 2 | 1 | NA | 50 | Jan-93 | Oct-10 | Apr-06 |
| 48 | D | 0 | 3 | 2 | 0 | NA | 36 | Jun-06 | Oct-10 | Nov-10 |
| 49 | B | 2 | 1 | 2 | 1 | 47 | 47 | Mar-98 | Oct-10 | Nov-09 |
| 50 | B | 0 | 3 | 1 | 1 | NA | 32 | None | Nov-08 | Jul-96 |
| 51 | B | 0 | 3 | 1 | 1 | NA | 163 | None | Dec-08 | Sep-92 |
| 52 | B | 0 | 3 | 1 | 1 | NA | 163 | None | Oct-06 | Jan-99 |
| 53 | D | 0 | 3 | 2 | 0 | NA | 48 | Jan-04 | Mar-11 | Jun-11 |
| 54 | B | 2 | 1 | 2 | 0 | 20 | 20 | May-06 | Mar-11 | Oct-11 |
| 55 | CRF01_AE | 0 | 3 | 2 | 0 | NA | 155 | Aug-09 | Apr-11 | Feb-06 |
| 56 | C | 0 | 3 | 2 | 0 | NA | 65 | Apr-09 | May-11 | Jul-08 |
| **Patient ID^A^** | **Clade^B^** | **Trans. Ev.^C^** | **Trans. Acc.^D^** | **Infect. Ev.^E^** | **Infect. Acc.^F^** | **Presumed Source^G^** | **Predicted Source^H^** | **Last Negative Date^I^** | **First Positive Date^J^** | **Pred. Infect. Date^K^** |
| 57 | CRF01_AE | 0 | 3 | 2 | 0 | NA | 144 | May-05 | Aug-11 | Sep-89 |
| 58 | CRF02_AG | 0 | 3 | 1 | 1 | NA | 148 | None | Jul-11 | Nov-96 |
| 59 | B | 0 | 3 | 1 | 1 | NA | 23 | None | Nov-07 | Nov-02 |
| 60 | C | 2 | 1 | 2 | 1 | 10 | 10 | Jun-10 | Sep-11 | Dec-10 |
| 61 | B | 1 | 3 | 2 | 0 | NA | 143 | Jan-09 | Nov-11 | Dec-12 |
| 62 | C | 0 | 3 | 1 | 1 | NA | 168 | None | Jan-12 | May-96 |
| 63 | B | 0 | 3 | 1 | 1 | NA | 170 | None | Oct-08 | Dec-92 |
| 64 | B | 1 | 2 | 1 | 0 | NA | 67 | None | Mar-12 | Feb-13 |
| 65 | C | 0 | 3 | 2 | 0 | NA | 150 | Mar-09 | Apr-12 | Aug-07 |
| 66 | A | 0 | 3 | 2 | 0 | NA | 13 | Sep-07 | Mar-12 | Aug-04 |
| 67 | NA | 2 | 0 | 2 | 0 | 64 | 147 | Jun-12 | Jun-12 | Jun-11 |
| 68 | B | 0 | 3 | 2 | 0 | NA | 51 | Nov-10 | Mar-11 | Jun-09 |
| 69 | B | 0 | 3 | 2 | 0 | NA | 135 | Jan-11 | Jan-12 | Jul-98 |
| 70 | C | 1 | 2 | 2 | 0 | NA | 71 | Jun-08 | Nov-12 | Dec-13 |
| 71 | C | 2 | 0 | 2 | 0 | 70 | 153 | Jan-12 | Nov-12 | Dec-06 |
| 72 | B | 0 | 3 | 2 | 0 | NA | 158 | Nov-11 | Mar-12 | May-88 |
| 73 | CRF07_BC | 1 | 3 | 2 | 0 | NA | 110 | May-11 | May-12 | Aug-07 |
| 74 | B | 0 | 3 | 2 | 0 | NA | 152 | Oct-10 | Apr-13 | Nov-03 |
| 75 | G | 1 | 2 | 0 | 3 | NA | 79 | None | None | Nov-11 |
| 76 | G | 2 | 1 | 2 | 0 | 75 | 75 | Jun-12 | Jan-13 | Apr-14 |
| 77 | B | 0 | 3 | 2 | 0 | NA | 166 | Oct-08 | Jun-13 | Jul-01 |
| 78 | A1 | 1 | 2 | 2 | 1 | NA | 98 | Jan-06 | Jul-13 | Feb-11 |
| 79 | G | 2 | 0 | 1 | 1 | 76 | 111 | None | May-13 | Jun-10 |
| 80 | C | 1 | 3 | 1 | 1 | NA | 121 | None | Aug-13 | Feb-10 |
| 81 | B | 2 | 0 | 2 | 0 | 37 | 160 | Jan-07 | Aug-13 | Mar-04 |
| 82 | B | 2 | 1 | 2 | 0 | 33 | 89 | Sep-12 | Aug-13 | May-14 |
| 83 | B | 2 | 1 | 1 | 1 | 81 | 81 | None | Aug-13 | Feb-13 |
| 84 | C | 2 | 1 | 1 | 1 | 80 | 80 | None | Oct-13 | Feb-12 |
| **Patient ID^A^** | **Clade^B^** | **Trans. Ev.^C^** | **Trans. Acc.^D^** | **Infect. Ev.^E^** | **Infect. Acc.^F^** | **Presumed Source^G^** | **Predicted Source^H^** | **Last Negative Date^I^** | **First Positive Date^J^** | **Pred. Infect. Date^K^** |
| 85 | C | 0 | 3 | 2 | 0 | NA | 86 | Aug-12 | Dec-13 | Jul-03 |
| 86 | C | 0 | 3 | 1 | 1 | NA | 168 | None | Dec-13 | Jun-91 |
| 87 | C | 2 | 1 | 2 | 0 | 73 | 73 | Dec-12 | Jan-13 | Sep-14 |
| 88 | AG | 0 | 3 | 1 | 1 | NA | 58 | None | Feb-14 | Mar-10 |
| 89 | B | 2 | 0 | 2 | 1 | 82 | 74 | Jan-11 | Mar-14 | Jul-13 |
| 90 | B | 2 | 1 | 2 | 0 | 17 | 17 | Jan-14 | Mar-14 | Jan-15 |
| 91 | B | 1 | 3 | 1 | 1 | NA | 164 | None | May-14 | Nov-02 |
| 92 | B | 2 | 1 | 2 | 1 | 91 | 91 | Jan-01 | May-14 | Feb-14 |
| 93 | CRF03_AB | 0 | 3 | 2 | 0 | NA | 104 | Feb-08 | Aug-09 | Jan-92 |
| 94 | C | 1 | 2 | 1 | 0 | NA | 105 | None | Feb-95 | Jan-10 |
| 95 | B | 0 | 3 | 2 | 0 | NA | 164 | Jan-14 | May-14 | Dec-00 |
| 96 | CRF01_AE | 0 | 3 | 2 | 0 | NA | 155 | Jan-07 | Mar-12 | Feb-01 |
| 97 | C | 0 | 3 | 2 | 0 | NA | 153 | Dec-12 | Nov-14 | Jun-97 |
| 98 | NA | 2 | 0 | 1 | 1 | 78 | 156 | None | Nov-14 | Feb-06 |
| 99 | AE | 0 | 3 | 1 | 1 | NA | 112 | None | Oct-14 | Dec-98 |
| 100 | B | 0 | 3 | 2 | 0 | NA | 11 | Jan-12 | Feb-15 | Apr-08 |
| 101 | B | 2 | 0 | 2 | 0 | 89 | 160 | Jul-14 | Feb-15 | Feb-96 |
| 102 | B | 2 | 1 | 2 | 1 | 61 | 61 | Sep-14 | Jun-15 | Mar-15 |
| 103 | B | 2 | 1 | 1 | 1 | 29 | 29 | None | Jun-15 | Jul-12 |
| 104 | B | 0 | 3 | 2 | 0 | NA | 172 | Jul-15 | Aug-15 | Dec-89 |
| 105 | C | 2 | 0 | 2 | 0 | 94 | 62 | Nov-14 | Sep-15 | Jul-00 |
| 106 | A / AE | 0 | 3 | 2 | 0 | NA | 57 | Jan-05 | Sep-15 | Apr-02 |
| 107 | B | 1 | 3 | 1 | 1 | NA | 27 | None | Oct-15 | Apr-13 |
| 108 | A | 2 | 1 | 2 | 0 | 12 | 12 | Apr-13 | Aug-15 | Apr-12 |
| 109 | B | 2 | 1 | 2 | 1 | 107 | 107 | Sep-13 | Nov-15 | Aug-15 |
| 110 | C | 0 | 3 | 2 | 0 | NA | 133 | Sep-14 | Sep-15 | Jun-99 |
| 111 | G | 0 | 3 | 2 | 0 | NA | 38 | Sep-10 | Dec-15 | May-97 |
| 112 | AE | 0 | 3 | 2 | 0 | NA | 57 | Sep-15 | Nov-15 | Feb-98 |
| **Patient ID^A^** | **Clade^B^** | **Trans. Ev.^C^** | **Trans. Acc.^D^** | **Infect. Ev.^E^** | **Infect. Acc.^F^** | **Presumed Source^G^** | **Predicted Source^H^** | **Last Negative Date^I^** | **First Positive Date^J^** | **Pred. Infect. Date^K^** |
| 113 | AG | 0 | 3 | 1 | 1 | NA | 148 | None | Apr-16 | Apr-10 |
| 114 | G | 0 | 3 | 1 | 1 | NA | 120 | None | Jun-16 | Aug-11 |
| 115 | B | 1 | 3 | 2 | 1 | NA | 141 | Jan-01 | Jun-16 | Nov-14 |
| 116 | B | 2 | 1 | 2 | 0 | 115 | 115 | Jan-14 | Jun-16 | Jan-17 |
| 117 | C | 0 | 3 | 2 | 0 | NA | 171 | Aug-13 | Aug-16 | Nov-90 |
| 118 | B | 0 | 3 | 2 | 0 | NA | 74 | Jul-16 | Aug-16 | Dec-10 |
| 119 | C | 0 | 3 | 2 | 0 | NA | 153 | Jan-14 | Sep-16 | Nov-05 |
| 120 | G | 0 | 3 | 2 | 0 | NA | 161 | Feb-16 | Oct-16 | Oct-03 |
| 121 | C | 0 | 3 | 1 | 1 | NA | 2 | None | Apr-17 | May-03 |
| 122 | B | 1 | 2 | 1 | 0 | NA | 123 | None | Apr-17 | Jul-17 |
| 123 | B | 2 | 0 | 2 | 1 | 122 | 146 | Jan-05 | Apr-17 | Jul-11 |
| 124 | C | 0 | 3 | 2 | 0 | NA | 162 | Jan-16 | Dec-16 | Jun-97 |
| 125 | B | 0 | 3 | 1 | 1 | NA | 158 | None | May-17 | Jan-06 |
| 126 | B | 0 | 3 | 0 | 3 | NA | 29 | None | None | Mar-11 |
| 127 | B | 1 | 2 | 2 | 0 | NA | 129 | Jan-15 | Jun-17 | Jun-18 |
| 128 | B | 0 | 3 | 2 | 0 | NA | 146 | Jan-15 | Jul-17 | Nov-93 |
| 129 | B | 2 | 0 | 1 | 1 | 127 | 128 | None | Jul-17 | Jun-17 |
| 130 | C | 2 | 1 | 2 | 0 | 16 | 16 | Sep-16 | Aug-17 | Dec-12 |
| 131 | B | 0 | 3 | 2 | 0 | NA | 35 | Oct-16 | Oct-17 | Mar-12 |
| 132 | AE | 0 | 3 | 2 | 0 | NA | 55 | Jul-17 | Oct-17 | Aug-16 |
| 133 | C | 0 | 3 | 2 | 0 | NA | 86 | Jan-14 | Oct-17 | Jul-94 |
| 134 | C | 0 | 3 | 1 | 1 | NA | 130 | None | Oct-17 | Dec-15 |
| 135 | B | 0 | 3 | 2 | 0 | NA | 147 | Sep-17 | Dec-17 | Aug-93 |
| 136 | A | 1 | 2 | 2 | 1 | NA | 137 | Jan-13 | Jan-18 | May-17 |
| 137 | A | 2 | 0 | 2 | 0 | 136 | 142 | Aug-16 | Jan-18 | Jul-13 |
| 138 | C | 0 | 3 | 2 | 0 | NA | 86 | Jan-17 | Jan-18 | Dec-93 |
| 139 | B | 0 | 3 | 1 | 1 | NA | 5 | None | Feb-18 | Jan-03 |
