# Supplementary material for: Evaluation of A Phylogenetic Pipeline to Examine Transmission Networks in A Canadian HIV Cohort
Source: Microorganisms. 2020 Jan 31;8(2):196. doi: 10.3390/microorganisms8020196 (PMC7074708; doi:10.3390/microorganisms8020196)
Supplement: Supplementary file 1 [file microorganisms-08-00196-s001.zip › Mak_ etal_Table_S2.docx]

| parameter Set | MCMC Operators | | | | | | | |
| --- | --- | --- | --- | --- | --- | --- | --- | --- |
|  | **Operates on** | | | **Parameter** | | | **Scale Factor** | **Weight** |
| Default | FrequenciesExchanger | | | DeltaExchangeOperator | | | 0.01 | 0.1 |
|  | StrictClockRateScaler | | | clockRate | | | 0.75 | 3.0 |
|  | strictClockUpDownOperator | | | clockRate | | | 0.75 | 3.0 |
|  | KappaScaler | | | kappa | | | 0.5 | 0.1 |
|  | CoalescentConstantTreeScaler | | | tree | | | 0.5 | 3.0 |
|  | CoalescentConstantTreeRootScaler | | | tree | | | 0.5 | 3.0 |
|  | CoalescentConstantUniformOperator | | | Uniform | | | NA | 30.0 |
|  | CoalescentConstantSubtreeSlide | | | SubtreeSlide | | | NA | 15.0 |
|  | CoalescentConstantNarrow | | | tree | | | NA | 15.0 |
|  | CoalescentConstantWide | | | tree | | | NA | 3.0 |
|  | CoalescentConstantWilsonBalding | | | WilsonBalding | | | NA | 3.0 |
|  | PopSizeScaler | | | popSize | | | 0.75 | 3.0 |
| Adjusted | FrequenciesExchanger | | | DeltaExchangeOperator | | | 0.01 | 0.1 |
|  | RateACScaler | | | rateAC | | | 0.5 | 0.1 |
|  | RateAGScaler | | | rateAG | | | 0.5 | 0.1 |
|  | RateATScaler | | | rateAT | | | 0.5 | 0.1 |
|  | RateCGScaler | | | rateCG | | | 0.5 | 0.1 |
|  | RateGTScaler | | | rateGT | | | 0.5 | 0.1 |
|  | ucldMeanScaler | | | ucldMean | | | 0.5 | 1 |
|  | ucldStdevScaler | | | ucldStdev | | | 0.5 | 3 |
|  | CategoriesRandomWalk | | | IntRandomWalkOperator | | | 1 | 10 |
|  | CategoriesSwapOperator | | | SwapOperator | | | NA | 10 |
|  | CategoriesUniform | | | UniformOperator | | | NA | 10 |
|  | relaxedUpDownOperator | | | UpDownOperator | | | 0.75 | 4 |
|  | BDSKY_serialtreeScaler | | | ScaleOperator | | | 0.5 | 3 |
|  | BDSKY_serialtreeRootScaler | | | ScaleOperator | | | 0.5 | 3 |
|  | BDSKY_serialUniformOperator | | | Uniform | | | NA | 30 |
|  | BDSKY_serialSubtreeSlide | | | SubtreeSlide | | | NA | 15 |
|  | BDSKY_serialnarrow | | | tree | | | NA | 15 |
|  | BDSKY_serialwide | | | tree | | | NA | 3 |
|  | BDSKY_serialWilsonBalding | | | WilsonBalding | | | NA | 3 |
|  | becomeUninfectiousRateScaler | | | ScaleOperator | | | 0.75 | 2 |
|  | samplingScaler | | | ScaleOperator | | | 0.75 | 2 |
|  | reproductiveNumberScaler | | | ScaleOperator | | | 0.75 | 10 |
|  | updownBD | | | UpDownOperator | | | 0.75 | 2 |
|  | origScaler | | | ScaleOperator | | | 0.75 | 1 |
|  | **PRIORS** | | | | | | | |
| Parameter set | **Parameter** | **Distribution** | **Initial Value** | | **Bound** | **Description** | | |
| DEFAULT | kappa | LogNormal | 2 | | [-∞, ∞] | HKY transition-transversion parameter | | |
|  | freqParameter | Uniform | 0.25 | | [0, 1] | base frequencies | | |
|  | clock.rate | Uniform | 1 | | [0, ∞] | substitution rate | | |
|  | constant.popSize | 1/X | 0.3 | | [-∞, ∞] | coalescent population size parameter | | |
| ADJUSTED | rateAC | Gamma | 1 | | [0, ∞] | GTR A-C substitution parameter | | |
|  | rateAG | Gamma | 1 | | [0, ∞] | GTR A-G substitution parameter | | |
|  | rateAT | Gamma | 1 | | [0, ∞] | GTR A-T substitution parameter | | |
|  | rateCG | Gamma | 1 | | [0, ∞] | GTR C-G substitution parameter | | |
|  | rateGT | Gamma | 1 | | [0, ∞] | GTR G-T substitution parameter | | |
|  | ucldMean | Uniform | 1 | | [-∞, ∞] | uncorrelated lognormal relaxed clock mean rate | | |
|  | ucldStdev | Gamma | 0.1 | | [0, ∞] | uncorrelated lognormal relaxed clock stdev | | |
|  | freqParameter | Uniform | 0.25 | | [0, 1] | base frequencies | | |
|  | reproductiveNumber | LogNormal | 2 | | [0, ∞] |  | | |
|  | samplingProportion | Beta | 0.01 | | [0, 1] |  | | |
|  | becomeUninfectiousRate | LogNormal | 1 | | [0, ∞] |  | | |
