# Supplementary material for: Evaluation of A Phylogenetic Pipeline to Examine Transmission Networks in A Canadian HIV Cohort
Source: Microorganisms. 2020 Jan 31;8(2):196. doi: 10.3390/microorganisms8020196 (PMC7074708; doi:10.3390/microorganisms8020196)
Supplement: Supplementary file 1 [file microorganisms-08-00196-s001.zip › Mak_ etal_Table_S3.docx]

**Supplementary Table 3:**

Summary statistics of parameter testing for TransPhylo Shape and Scale parameter testing for infection and sampling rate. The number of correct predictions for transmissions and infection dates over a hundred runs was used to determine the best combination of parameters.

| **Combination** | **Avg Correct Date Predictions** | **Avg of Correct Transmission Predictions** |
| --- | --- | --- |
| Shape_ 1 _Scale_ 1 | 24.33 | 16.35 |
| Shape_ 1 _Scale_ 1.1 | 24.3 | 17.06 |
| Shape_ 1 _Scale_ 1.2 | 23.73 | 16.41 |
| Shape_ 1 _Scale_ 1.3 | 23.74 | 16.89 |
| Shape_ 1 _Scale_ 1.4 | 23.14 | 16.82 |
| Shape_ 1 _Scale_ 1.5 | 23.69 | 16.64 |
| Shape_ 1 _Scale_ 1.6 | 23.02 | 16.35 |
| Shape_ 1 _Scale_ 1.7 | 23.06 | 16.91 |
| Shape_ 1 _Scale_ 1.8 | 23.69 | 16.85 |
| Shape_ 1 _Scale_ 1.9 | 22.6 | 17.23 |
| Shape_ 1 _Scale_ 2 | 22.51 | 16.65 |
| Shape_ 2 _Scale_ 1 | 23.78 | 16.93 |
| Shape_ 2 _Scale_ 1.1 | 23.6 | 17.22 |
| Shape_ 2 _Scale_ 1.2 | 23.25 | 17.17 |
| Shape_ 2 _Scale_ 1.3 | 22.85 | 16.9 |
| Shape_ 2 _Scale_ 1.4 | 22.84 | 17.56 |
| Shape_ 2 _Scale_ 1.5 | 22.59 | 17.94 |
| Shape_ 2 _Scale_ 1.6 | 21.92 | 16.86 |
| Shape_ 2 _Scale_ 1.7 | 21.93 | 17.9 |
| Shape_ 2 _Scale_ 1.8 | 21.65 | 17.83 |
| Shape_ 2 _Scale_ 1.9 | 21.23 | 17.67 |
| Shape_ 2 _Scale_ 2 | 21.24 | 17.74 |

TransPhylo adapts gamma distributions for the sampling time and generation time distributions. The shape and scale represent the parameters of these gamma distributions.
