# Supplementary material for: Evaluation of A Phylogenetic Pipeline to Examine Transmission Networks in A Canadian HIV Cohort
Source: Microorganisms. 2020 Jan 31;8(2):196. doi: 10.3390/microorganisms8020196 (PMC7074708; doi:10.3390/microorganisms8020196)
Supplement: Supplementary file 1 [file microorganisms-08-00196-s001.zip › Mak_etal_2019_Supplementary_Materials.docx]

**Supplementary Material**

**Methods**

**The SAC ‘Gold Standard’ Dataset:**

The gold standard’ dataset was composed of patients with a strong epidemiologic link as indicated by documented blood-borne transmission [1], congenital infection, public health contact tracing, legal proceedings or self-reporting.

**Phylogenetic Tree Generation:**

The quality of the BEAST-based tree generation process was analyzed by Tracer [2] (<http://beast.community/tracer>), and a maximum-clade credibility (MCC) tree was generated by TreeAnnotator in Newick format [3] (<http://beast.community/treeannotator>).

The adjusted model was selected specifically because it was built for the analysis of serially sampled infected populations of rapidly evolving RNA viruses such as the human immunodeficiency virus and the hepatitis C virus [4]. The exact values parameterizing the adjusted models was set as suggested by the designers of the birth-death skyline serial model in the tutorial accompanying the paper [5].

**Transmission Relationship and Infection Date Inference**:

The likelihood that a source patient (one who has transmitted to others) is sampled is a measure of how unlikely TransPhylo is to insert an unsampled patient into the tree. The greater the likelihood, the fewer unsampled patients TransPhylo inserts into the tree. The generation time distribution describes the time between a patient becoming infected and infecting another patient in the tree. The sampling distribution describes the time between a patient becoming infected and having a sample drawn at SAC. Both distributions are gamma distributions, which models the time required for *m* events to occur in a Poisson process with mean incidence time of n, where *m* = shape parameter and 1 / *n* = scale parameter. Many combinations of the initial shape and rate parameters were attempted to better approximate the within-patient infection dynamics of HIV and the window of time during which a patient is generally contagious (i.e.: when the viral load is not suppressed).

The fixedness of a parameter refers to the treatment of the parameter during the Markov chain Monte Carlo (MCMC) inference process. Briefly, each iteration of the MCMC inference process updates the value of a parameter by drawing from a range of possible values, which is determined by the type of the parameter’s distribution. If a parameter is treated as fixed, it is not updated at all by MCMC. If a parameter is unfixed, its value will be updated throughout the inference process. The updates are made such that, in general, the value gets closer and closer to one that best reflects the epidemiological conditions. For example, if the likelihood that a source is sampled is left unfixed, and it is initiated as 0.5, the value 0.5 will be gradually adjusted through the inference process such that the end-value is closer to the actual likelihood of being sampled. In our attempts, the likelihood ended up as 0.0273 on average. This is reflected in the large number of predicted unsampled patients in transmission trees made with default TransPhylo parameters (**Table 2**).

**Summary Statistics of the Transmission Tree:**

There are seven summary statistics in total: i) the total number of patients, sampled and unsampled, represented in the tree; ii) the number of sampled patients predicted to be infected after 1989, iii) the number of sampled patients predicted to be infected after 1989 infected by another sampled patient, iv) the number of unsampled patients predicted to be infected after 1989, v) the proportion of iii) relative to ii), vi) the proportion of iv) relative to the sum of ii) and iv), and vii) the ratio of patients who infected two or more other patients to those who only infected one other.

For example, let there be 100 patients in a TransPhylo-generated transmission tree (i). Let there be 80 sampled patients (ii) out of that 100 predicted to be infected after 1989. Sampled means that the SAC is aware of them and has biobanked samples from them. Of those 80, let there be 20 (iii) who were also infected by another sampled patient infected after 1989.

There are also 10 unsampled patients (iv) predicted to be infected after 1989. These are patients that TransPhylo has inferred the presence of. As a corollary, these 10 patients are either 1) unknown to the SAC entirely or 2) known, but are not being clinically treated. Ergo, these patients are an estimate of the lost-to-follow-up (LTFU) or unknown positive population in the southern Alberta region. The proportion of these patients relative to the monitored HIV-positive population is 10/80 (vi).

The proportion of sampled patients infected after 1989 relative to all patients in the tree is 80/100, or 0.80 (v). This is a measure of the proportion of new infections that arose due to local expansion of transmission clusters, as opposed to new infections from individuals moving to southern Alberta with pre-existing infections.

The ratio of patients who infected two or more other patients to those who only infected one other was calculated from the raw data.

**Scripts:**

Custom R scripts were written to extract summary statistics about the topology of the inferred transmission trees and extract inferred person-to-person (P2P) relationships. A custom Python script was written to compare the inferred P2P transmission relationships and infection dates to the clinically known information. Both can be found at https://github.com/theLongLab/Transmission_Analyzer.

**Results**

**Validation of Phylogenetic Methods with Clinical Epidemiology**: Out of 28 patients with complete transmission relationship and infection date range information, 6 were completely and correctly inferred. Thus, 22/28 transmission relationships were correctly inferred despite incorrectly inferred infection dates.

**Transmission Tree Analysis**: The predicted transmission tree in Figure 4 was generated from adjusted BEAST and adjusted TransPhylo parameters and predicted the largest number of accurate person-to-person (P2P) transmission relationships. The tree was rooted by a most recent common ancestor HIV sequence (MRCA) just before the 1980s, which i) was consistent with the oldest HIV-1 group M genomes extracted from patient samples in 1978-1979 [7]. However, it is important to note that ii) the tree represents the MRCA of multiple HIV subtypes, which developed in Africa much earlier than the 1970’s [7] and iii) the tree was made with the assumption of non-uniform substitution rates on each branch. Though the split of HIV subtypes was, in most cases, much older than 1970, assumption (iii) reconciles the different root-times that would be expected given (i) and (ii), which is was analogous to a weighted average of the effects of (i) and (ii). A similar tree made with the default BEAST parameters, which required uniform substitution rates on each branch, the MRCA was dated in the 1910's (Supp. Figure 1), which agrees with most modern estimates of the HIV subtype ancestor [8].

**Infection Date Inference**: Of the 87 patients with fully bounded infection date ranges, 13 patients had predicted infection dates that fell within their ranges. Of the 74 that did not, 56 were predicted to be before the LN, and 18 were predicted to be after the FP. As mentioned in the main text, the TransPhylo adjusted parameters demonstrated a tendency to back-date the time of predicted infection. For comparison, the replicate where 30 infection dates were accurately inferred was also compared to determine whether this back-dating phenomenon was unique to the transmission-aware TransPhylo model. This replicate was generated by MUSCLE, adjusted BEAST parameters, and default TransPhylo parameters (**Table S2**). 30 patients had predicted infection dates that fell within their fully bounded ranges. Of the 57 patients that did not, 26 were predicted to be before the LN, and 31 were predicted to be after the FP. Despite the slight tendency to forward-date the time of infection, this is much closer to random prediction error that is expected occur if the transmission inference model was not biased for older infection dates. However, the number of accurately recovered transmission relationships was extremely low, 8/36. This again demonstrates the transmission relationship-infection date tradeoff.

**SUPPLEMENTARY FIGURE AND TABLE CAPTIONS**

**Figure S1:** Transmission tree generated by HIVAlign, default BEAST parameters, and adjusted TransPhylo parameters. Sampled patients are represented by black branches, and unsampled patients by light grey branches. The symbols represent person-to-person transmission events. Green and red triangles represent correctly and incorrectly inferred relationships respectively. Grey squares represent novel inferences of transmission relationships that have no precedence in the SAC dataset.

**References**

1. Gill MJ, Towns D, Allaire S, Meyers G. Transmission of human immunodeficiency virus through blood transfusion: the use of lookback and trackback approaches to optimize recipient identification in a regional population. Transfusion. **1997**;37:513-516
2. Rambaut A, Drummond AJ, Xie D, Baele G, Suchard MA. Posterior summarisation in Bayesian phylogenetics using Tracer 1.7. Syst. Biol. **2018** Apr 27;10.
3. Rambaut A, Drummond AJ, 2013. TreeAnnotator v1.7.0. Available as part of the BEAST package at http://beast.bio.ed.ac.uk. **2013**.
4. Stadler T, Kühnert D, Bonhoeffer S, Drummond AJ. Birth–death skyline plot reveals temporal changes of epidemic spread in HIV and hepatitis C virus (HCV). Proceedings of the National Academy of Sciences. **2013** Jan 2;110(1):228-33.
5. Kuhnert, D, 2015. Birth–Death skyline (BDSKY) tutorial. [ebook] Available at: https://www.beast2.org/files/2015/05/bdskytutorialv2.1.1.pdf [Accessed 5 Sep. 2018].
6. Krentz HB, John Gill M. Long-term HIV/AIDS survivors: Patients living with HIV infection retained in care for over 20 years. What have we learned? International Journal of STD & AIDS. **2018** Jun 22:0956462418778705.
7. Worobey M, Watts TD, McKay RA, Suchard MA, Granade T, Teuwen DE, Koblin BA, Heneine W, Lemey P, Jaffe HW. 1970s and ‘Patient 0’HIV-1 genomes illuminate early HIV/AIDS history in North America. Nature. **2016** Nov;539(7627):98.
8. Korber B, Muldoon M, Theiler J, Gao F, Gupta R, Lapedes A, Hahn BH, Wolinsky S, Bhattacharya T. Timing the ancestor of the HIV-1 pandemic strains. science. **2000** Jun 9;288(5472):1789-96.
