# Supplementary figures and images for: Evaluation of A Phylogenetic Pipeline to Examine Transmission Networks in A Canadian HIV Cohort
Source: Microorganisms. 2020 Jan 31;8(2):196. doi: 10.3390/microorganisms8020196 (PMC7074708; doi:10.3390/microorganisms8020196)

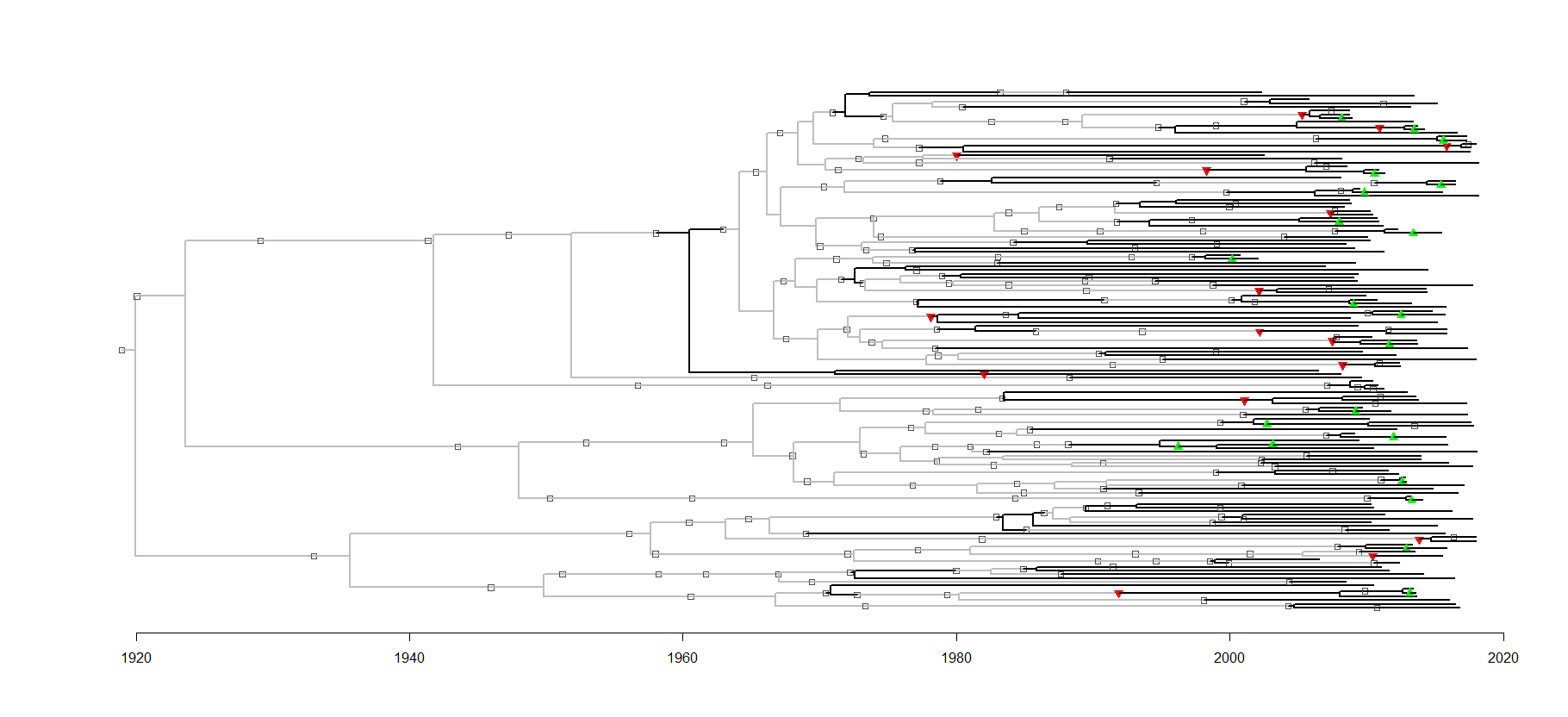

Supplement: Supplementary file 1 [file microorganisms-08-00196-s001.zip › Figure_S1.png]
